# Identification of miR-29c-3p as a Robust Normalizer for Urine microRNA Studies in Bladder Cancer

**DOI:** 10.3390/biomedicines8110447

**Published:** 2020-10-22

**Authors:** Julia Oto, Emma Plana, Álvaro Fernández-Pardo, Fernando Cana, Manuel Martínez-Sarmiento, César D. Vera-Donoso, Francisco España, Pilar Medina

**Affiliations:** 1 Haemostasis, Thrombosis, Arteriosclerosis and Vascular Biology Research Group, Medical Research Institute Hospital La Fe, 46026 Valencia, Spain; juliaotomartinez@gmail.com (J.O.); plana_emm@gva.es (E.P.); alvarofernandezpardo@gmail.com (Á.F.-P.); fernandocana1998@gmail.com (F.C.); espanya_fra@gva.es (F.E.); 2 Angiology and Vascular Surgery Service, La Fe University and Polytechnic Hospital, 46026 Valencia, Spain; 3 Department of Urology, La Fe University and Polytechnic Hospital, 46026 Valencia, Spain; mmarsar@gmail.com (M.M.-S.); cdveradonoso@gmail.com (C.D.V.-D.)

**Keywords:** bladder cancer, urine, miRNA, reference miRNA, normalizer, RT-qPCR

## Abstract

Bladder cancer (BC) is among the most frequent malignancies worldwide, being the most expensive cancer to treat and monitor and the most lethal urological cancer. Urine microRNAs (miRNAs) have been proposed as novel non-invasive biomarkers to early diagnose and monitor BC patients in order to avoid the performance of current aggressive diagnostic techniques. However, huge discrepancies arise among studies mainly due to the lack of standardization in the normalization, a crucial step in all miRNA studies. Our aim was to identify the best miRNA normalizer for miRNA studies in urine of BC patients. We evaluated the performance of 110 candidate miRNAs in urine of 35 BC patients and 15 healthy controls by Real Time quantitative Polymerase Chain Reaction (RT-qPCR) followed by a stability analysis with RefFinder. In this screening stage, miR-29c-3p arose as the most stably expressed miRNA in BC and controls, with a good expression level. Stability of miR-29c-3p expression was validated in an independent cohort of 153 BC patients and 57 controls. Finally, we evaluated the robustness of miR-29c-3p as normalizer in the expression study of miR-200c-3p, a potential diagnostic marker for BC. We propose miR-29c-3p as a normalizer for miRNA studies in BC urine. This is the first study that characterizes a reliable normalizer that may allow the comparison of future urine miRNA studies as non-invasive biomarkers for BC diagnosis and monitoring.

## 1. Introduction

Bladder cancer (BC) is among the most frequent malignancies worldwide. Indeed, BC accounts for 3% of all malignant tumors in adults and is the most lethal urological tumor type. According to the GLOBOCAN 2018 study, BC accounts for more than 540,000 new cases worldwide and almost 200,000 deaths annually [1,2]. Moreover, BC largely increases health expenses since it is the most expensive cancer to treat and the one that accounts for the highest monitoring costs. In fact, the cost of the muscle-invasive subtype reaches $150,000 per capita [3,4] and, by the end of the decade, BC is expected to account for >3% of all cancer-related medical expenses [3].

Bladder ultrasound, computerized tomography (CT) scan, cystoscopy or cytology are presently the gold standard techniques for BC diagnosis. However, cystoscopy is a highly invasive procedure that causes a high discomfort in patients and urine cytology, while being non-invasive, is unable to detect low-grade bladder tumors. Furthermore, none of both achieve a high sensitivity and specificity [5]. These last two techniques are routinely used as follow-up methods. As a consequence, novel non-invasive biomarkers are being explored to early and accurately diagnose and monitor BC patients in order to avoid the performance of this aggressive technique while reaching or even exceeding the sensitivity and specificity of cystoscopy [6]. Urine represents the most accessible source of biological markers for the analysis of BC and other urological tumors since it is rapid and easily obtained by the patient, avoids patient discomfort and potential complications from an invasive procedure, is available in copious amounts and re-sampling is easily achievable [7]. Thus, urine represents the ideal sample to develop novel tools to diagnose and monitor the prognosis of BC patients.

microRNAs (miRNAs) are small non-coding RNAs that regulate protein expression. They have been found to play a role in the different processes of tumor development [8,9] and have been proposed as regulatory molecules and biomarkers in virtually all cancer types [10,11,12]. miRNAs are known to be released from tissues into biological fluids such as urine and have been proposed as biomarkers for many disorders. Indeed, several profiles of miRNAs have been proposed as diagnostic and prognostic tools for BC [13,14,15,16,17,18,19,20,21,22,23,24]. However, strikingly only a small number of miRNAs are shared by these studies, what is probably due to the lack of standardization in the protocols used among laboratories. Particularly results are often not reproducible among publications because of different criteria used for selecting patients and controls, the use of different procedures for sample processing, RNA isolation and miRNA quantification but, mainly, it may be caused by different normalization strategies used, what certainly represents a crucial step in any miRNA study. Several RNA species have been proposed as normalizers for miRNAs studies: small nuclear RNAs (snRNAs), nucleolar RNAs (snoRNAs), ribosomal RNAs (rRNAs), miRNAs or even exogenous synthetic RNAs. However, some of these proposed normalizers differ from miRNAs in length (150 nt for snRNAs and 60–200 nt for snoRNAs, compared to 20–24 nt for miRNAs) and also in structure [25]. This disparity in lengths and structure can affect the isolation efficiency, reverse transcription and amplification. Consequently, the use of stably expressed miRNAs for the normalization of miRNAs studies is the best option rather than the use of other small RNA species [26]. Likewise, the addition of exogenous synthetic RNAs is intended to track the isolation and reverse transcription efficiency, not to normalize for amplification. To date, no consensus exists on internal reference miRNAs for BC studies performed in urine samples by Real Time quantitative Polymerase Chain Reaction (RT-qPCR)

In the present study, we aimed for the first time to ascertain the best miRNA normalizer for miRNA studies in BC performed in urine samples. The discovery of a good and reproducible internal miRNA normalizer will eliminate the current inconsistency among studies and will finally allow the comparison of results obtained in urine of BC patients. This is an inexorable requirement in order to apply this technique to clinical practice.

## 2. Experimental Section

### 2.1. Study Subjects

**Figure 1:**
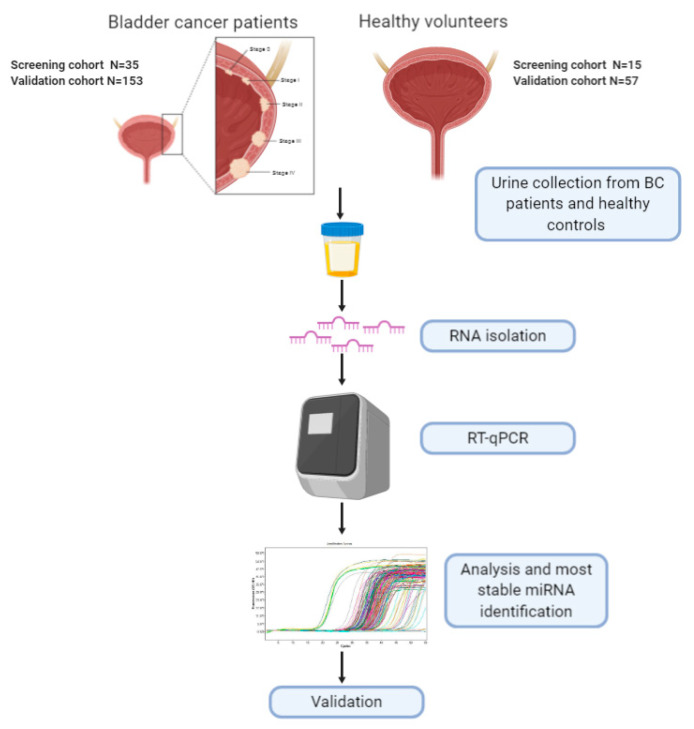
Graphical description of BC subtypes and study workflow.

BC can be subdivided in two types, superficial or non-muscle-invasive bladder cancer (NMIBC) (70% of total), which comprehends Ta and T1 lesions, and muscle-invasive bladder cancer (MIBC) (30% of total), which comprehends T2, T3 and T4 lesions. Additionally, grading indicates the degree of cellular differentiation, being G1 well differentiated and less likely to spread, G2 moderately differentiated and G3 poorly differentiated and more likely to spread. This can be better envisaged in Figure 1, where the study workflow is also described.

For the initial screening stage, 35 BC patients (10 TaG1, 8 TaG3, 5 T1G3 and 12 T2G3) were recruited at La Fe University and Polytechnic Hospital (Valencia, Spain). Fifteen healthy volunteers (control group) with similar age and sex who underwent an ultrasound scan to rule out the presence of urological malignancies or other alterations were also recruited. For the validation stage, an independent cohort of 153 BC patients (33 TaG1, 13 TaG3, 29 T1G3, 54 TaG2, 9 T1G2, 4 T2G2, 10 T2G3 and 1 T3G3) and 57 healthy controls were additionally recruited.

Pre-operative clinical staging was performed through physical examination, urine cytology and CT scans of the chest, abdomen and pelvis. The tumor histological classification was done according to both the 1973 and 2004/2016 WHO classifications. Demographic and clinical data were collected.

The exclusion criteria were lack of informed consent, absence of histological confirmation and presence of other malignancies.

Informed consent was obtained from all participants according to protocols approved by the ethics review board at La Fe University and Polytechnic Hospital (2014/0314 and 2017/0474). The study was performed according to the declaration of Helsinki, as amended in Edinburgh in 2000.

### 2.2. Urine Collection

A first morning urine sample of 25 to 50 mL was collected in sterile containers from all participants and kept at 4 °C until processing. Urine was centrifuged at 805× *g* for 5 min at 4 °C to remove cellular debris and supernatant was aliquoted and frozen at −80 °C until analyzed. The concentration of creatinine in urine was measured by clinical laboratory standardized methods.

### 2.3. RNA Isolation and cDNA Synthesis from Urine

Total urine RNA (including miRNAs) was isolated from 600 µL of urine using the miRNeasy Mini Kit (Qiagen, Hilden, Germany) following manufacturer’s instructions with several modifications optimized by our group [27]. Briefly, 200 µL of cell-free urine were transferred to a tube with 1 mL Qiazol (Qiagen) and 1 µL carrier (1 µg/µL yeast RNA, Invitrogen, ThermoFisher Scientific, Waltham, MA, USA). This step was done in three independent tubes for each sample. In one of the three tubes 1 µL spike-in mix (UniSp2/4/5, Exiqon, Vedbaek, Denmark) was also added and each tube was gently mixed. After a 5 min incubation at room temperature, 200 µL chloroform were added to each tube, and centrifuged at 12,000× *g*, 15 min at 4 °C to allow phase separation. Ethanol in a proportion of 1.5:1 (volume:volume) was added to the liquid phase. The three tubes containing the urine sample from the same individual were pooled in one single column in order to increase the final RNA yield. Then, 4 cleaning steps with the buffers supplied in the kit were performed. Total RNA was finally eluted in 50 µL of DNase/RNase-free sterile distilled water.

The concentration and purity of the RNA was assessed by spectrophotometric quantification with the NanoDrop ND-1000 (Thermo Fisher Scientific). RNA was stored at −80 °C until used.

In the initial screening stage where predesigned panels were used, cDNA was obtained from 5 µL of urine RNA with the miRCURY LNA RT Kit (Qiagen) according to the supplied protocol in a final reaction volume of 25 µL. Due to the addition of a RNA carrier during the isolation, the final RNA yield includes the RNA isolated from urine plus the carrier RNA. Therefore, urine RNA retrotranscription was based on volume (µL) rather than RNA quantity (ng), according to the suppliers’ recommendations. In the validation stage, where the expression level of selected miRNAs was conducted, cDNA was obtained from 2 µL of urine RNA using the same technology (final reaction volume 10 µL). In all cases, the reaction mix containing RNA, enzyme, buffer, RNAse-free water and UniSp6 RNA spike-in template, was incubated 60 min at 42 °C followed by 5 min at 95 °C for reverse transcriptase inactivation. Reactions were carried out in a thermocycler TC-412 (Techne, Minneapolis, MN, USA).

### 2.4. miRNAs Quantification

In the screening stage, 35 urine cDNA samples from BC patients and 15 from healthy controls were analyzed. In them, a total of 179 miRNAs were quantified using the commercially predesigned Serum/plasma miRNA PCR Panel V5 (Qiagen). This panel contains 179 miRNAs commonly found in human plasma and serum according to the manufacturer’s in-house analyses of miRNA expression in blood, serum and plasma samples, as well as on the limited number of peer-reviewed published papers available. The list of all the quantified miRNAs is detailed in Table S1. miRCURY LNA SYBR Green Master Mix (Qiagen) was used as a fluorophore, according to manufacturer´s indications. Briefly, cDNA (dilution 1/40), water and PCR master Mix (which includes SYBR Green) were added to a 384-well PCR plate supplied that includes the LNA^TM^ primer sets in a final reaction volume of 10 µL. Furthermore, each panel included the following internal controls: 5 synthetic RNAs of the RNA Spike-in kit aimed to monitor the RNA isolation and cDNA synthesis, and an inter-plate calibrator in triplicate and a negative control to evaluate qPCR performance. qPCR reactions were performed as follows: a polymerase activation/denaturation cycle of 2 min at 95 °C followed by 55 cycles of 10 s at 95 °C and 1 min at 56 °C with a ramp-rate of 2.2 °C/s. All RT-qPCR reactions were conducted in a LightCycler 480 II (Roche, Mannheim, Germany). In the validation stage, selected miRNAs were quantified using specific LNA PCR primer sets (Qiagen) in a total of 153 urine samples from BC patients and 57 healthy controls.

### 2.5. Selection of Candidate miRNA Normalizers and Analysis of Their Stability

To normalize the expression level of each miRNA, the best candidate with the highest stability and the lowest biological variance over the entire range of samples being investigated (BC and controls) was selected. To that aim, all miRNAs with a mean Ct < 35 in BC and controls were scrutinized. To select the best normalizer, the comprehensive tool RefFinder was employed which integrates the computational programs Genorm [28], BestKeeper [29], the comparative Delta Ct method [30] and NormFinder [31] (https://www.heartcure.com.au/for-researchers/) [32].

### 2.6. Statistical Analysis

Continuous variables were presented as median and interquartile range, and categorical variables as count and percentage. The analysis of variance (ANOVA) with the Tuckey Post Hoc test and unpaired t-test were used to identify significant differences in miRNA expression levels between BC patients and healthy controls. The statistical analysis was performed using the GraphPad Prism software v.8.0.1 (GraphPad software Inc., La Jolla, CA, USA). The Venn diagram was performed using the online tool available on http://bioinformatics.psb.ugent.be/webtools/Venn/. *p*-Values < 0.05 were considered statistically significant.

## 3. Results

### 3.1. Clinical Characteristics of the Study Subjects

A total of 188 BC patients were prospectively recruited together with 72 healthy volunteers (control group) with similar age and sex. The clinical characteristics of the study subjects are depicted in Table 1. The patients studied in the screening stage (*n* = 35) were: 10 patients (28.57%) with a TaG1 BC, 8 patients (22.86%) with TaG3, 5 patients (14.29%) with T1G3 and 12 patients (34.29%) with T2G3. The patients studied in the validation stage (*n* = 153) were: 33 patients (21.57%) with TaG1, 13 patients (8.5%) with TaG3, 29 patients (18.95%) with T1G3 and 15 patients (9.80%) with T2G2/T2G3/T3G3. Additionally, in order to validate the proposed normalizer in the whole spectrum of BC patients, 2 more groups of patients were included in the validation stage: TaG2 (54 patients, 35.29%) and T1G2 (9 patients, 5.88%).

### 3.2. Quality Internal Control with Synthetic Spike-in RNAs

**Figure 2:**
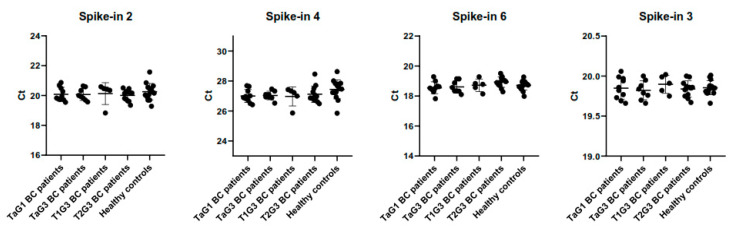
Differences in expression levels of three synthetic spike-in RNAs among BC patients with different stages and grades and healthy controls. Spike-in 2 and spike-in 4 monitor the RNA isolation step, spike-in 6 monitors the retrotranscription efficiency and spike-in 3 functions as inter-plate calibrator. Expression levels are represented as Ct values and error bars represent standard deviation.

To ensure that miRNA quantification was not influenced by technical and interpersonal variability, synthetic non-human spike-in RNAs are frequently used. We assessed the RNA isolation step by adding the synthetic spike-in 2 and spike-in 4 RNAs during all RNA isolations, and the retrotranscription efficiency by adding the spike-in 6 RNA in all retrotranscription reactions. No differences were observed in any spike-in studied among the study groups (Figure 2), thus indicating a proper performance of isolation and retrotranscription steps. To evaluate the qPCR performance of all reactions, the inter-plate calibrator spike-in 3 RNA in triplicate and a negative control were included in each panel. No differences were observed in any comparison made.

### 3.3. Selection of Candidate miRNA Normalizers and Analysis of Their Stability

**Figure 3:**
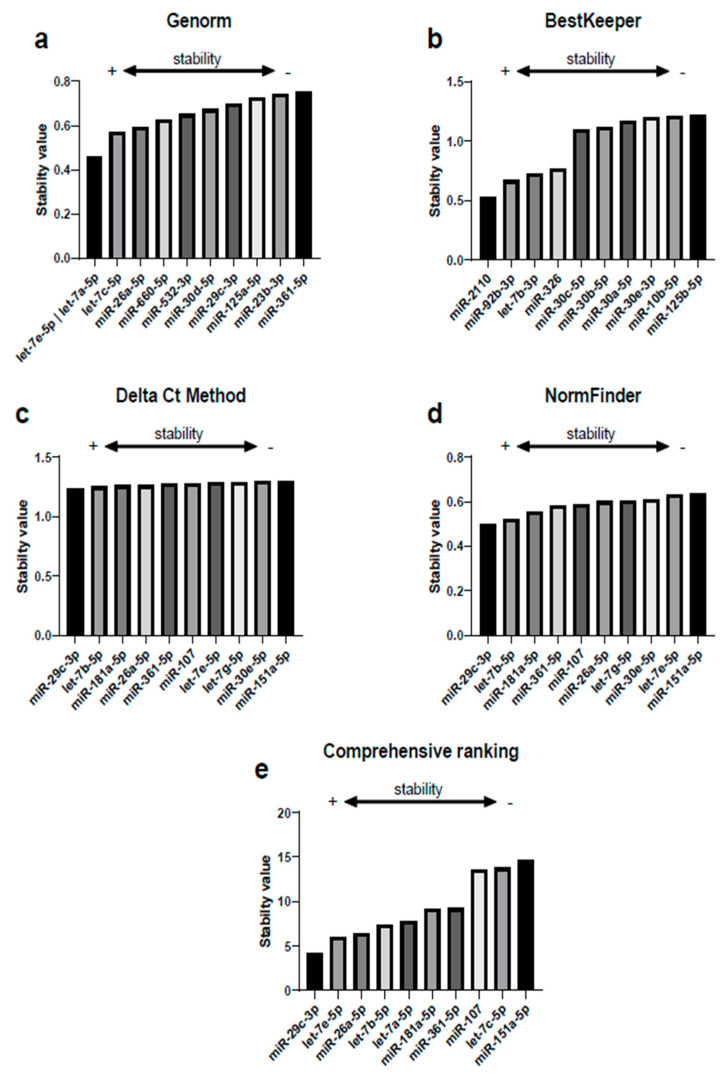
Selection of candidate miRNA normalizers and analysis of their stability conducted with the comprehensive tool RefFinder. Each graph represents the best 10 reference miRNAs selected by each algorithm: (**a**) Genorm, (**b**) BestKeeper, (**c**) Delta Ct method, (**d**) NormFinder and (**e**) Comprehensive ranking. The lower the stability value, the higher the stability of each miRNA.

**Figure 4:**
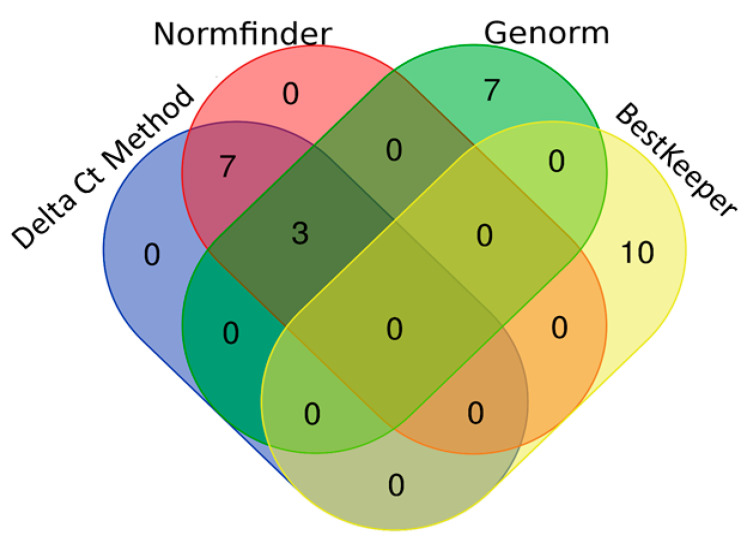
Venn diagram presenting the overlap among the best 10 reference miRNAs selected by each algorithm. Delta Ct method, NormFinder, Genorm and BestKeeper.

Of the 179 miRNAs quantified in each sample of the screening stage, we obtained high quality signals in 110 miRNAs (mean of the Ct < 35) both in BC patients and controls, thus they were included in the analysis with RefFinder. This tool comprehends the computational algorithms Genorm, BestKeeper, Delta Ct and NormFinder. Figure 3 shows the best 10 reference miRNAs selected by each algorithm. The stability analysis conducted with Genorm revealed that the greatest stability was reached by the combination of let-7e-5p and let-7a-5p (Figure 3a). BestKeeper revealed that the most stable miRNA was miR-2110 (Figure 3b). The Delta Ct method and NormFinder agreed with the most stable miRNA being miR-29c-3p (Figure 3c,d). Finally, the recommended comprehensive ranking that integrates all the previous analyses, rendered miR-29c-3p as the most stable miRNA (Figure 3e), being aligned with the results of the Delta Ct method and NormFinder. Next, we represented in a Venn diagram the overlap among the best 10 reference miRNAs selected by each algorithm (Figure 4). Three miRNAs were shared by the Delta Ct method, Genorm and NormFinder: miR-29c-3p, miR-26a-5p and miR-361-5p. Likewise, 7 miRNAs were shared by Delta Ct Method and NormFinder. In contrast, none of the 10 most stable miRNAs rendered by BestKeeper were selected by any other algorithm, thus none of them were included in the comprehensive ranking provided by RefFinder and consequently discarded as potential normalizers in our study.

### 3.4. Differences in Expression Levels of Candidate miRNA Normalizers between BC Patients and Controls

**Figure 5:**
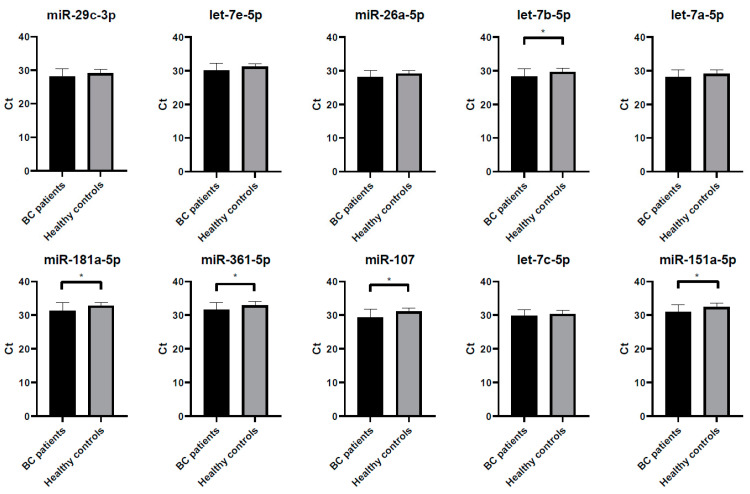
Differences in expression levels of the candidate miRNA normalizers selected by the comprehensive ranking of RefFinder between BC patients and controls. Expression levels are represented as Ct values and error bars represent standard deviation. Unpaired T-test: * *p* < 0.05.

A crucial characteristic of a good normalizer in miRNAs studies is the stable expression among the samples analyzed. Thus, we compared the mean Ct values of the best 10 reference miRNAs selected by the comprehensive ranking of RefFinder between BC patients and controls. No significant differences were observed in the expression of miR-29c-3p, let-7e-5p, miR-26a-5p, let-7a-5p and let-7c-5p (unpaired t-test, *p* > 0.05). In contrast, we found significant differences in the expression levels of let-7b-5p (*p* = 0.029), miR-181-5p (*p* = 0.026), miR-361-5p (*p* = 0.025), miR-107 (*p* = 0.012) and miR-151-5p (*p* = 0.015) (Figure 5). As expected, those miRNAs with the highest stability value had similar expression levels between BC and controls.

### 3.5. Effect of Different Normalization Strategies on the Relative Quantification of a miRNA Closely Related with BC

**Figure 6:**
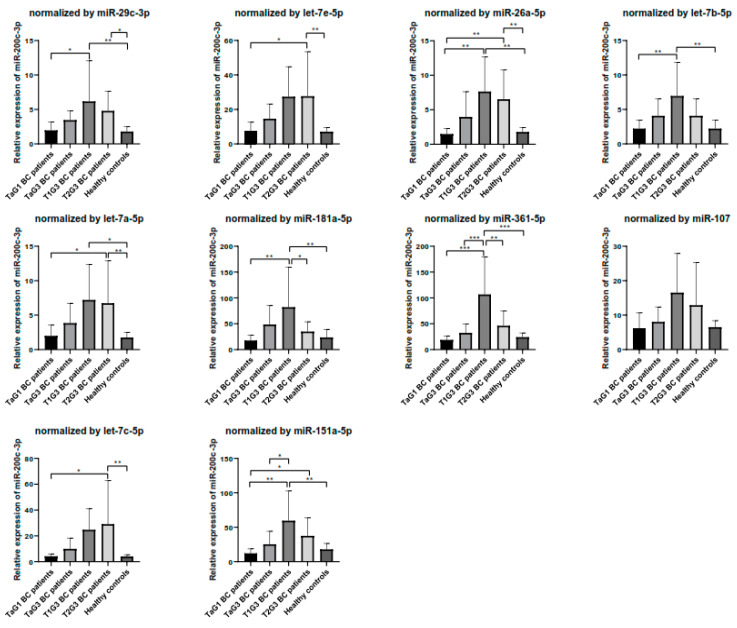
Relative expression of miR-200c-3p normalized by each candidate miRNA selected by the comprehensive ranking of RefFinder. Normalization was performed by the 2^−∆∆Ct^ method. Error bars represent the standard error of the mean. ANOVA with the Tuckey Post Hoc test: * *p* < 0.05; ** *p* < 0.01; *** *p* < 0.001.

miR-200c-3p has been previously proposed as urinary diagnostic biomarker for BC [24,33]. Thus, we evaluated the performance of the 10 most stable miRNAs, selected by the comprehensive ranking of RefFinder, as normalizers for miR-200c-3p quantification in the screening cohort. As seen in Figure 6, significant differences among the BC groups studied and controls were observed when any of the normalizers were used, although the results obtained were not always comparable in magnitude and trend.

In consideration of the aforementioned results, we selected miR-29c-3p as the best normalizer for the following reasons: (1) It was proposed as the most stable miRNA in the comprehensive analysis of RefFinder, and in two of the main algorithms independently tested (Figure 2); (2) Its expression level was optimal for a proper quantification (mean Ct = 28.4) (data not shown); and (3) Significant differences in the expression of miR-200c-3p, a miRNA related to BC, were observed when it was used as normalizer (Figure 6).

### 3.6. Validation of the Performance of miR-29c-3p as Normalizer in an Independent Cohort of BC Patients and Controls

**Figure 7:**
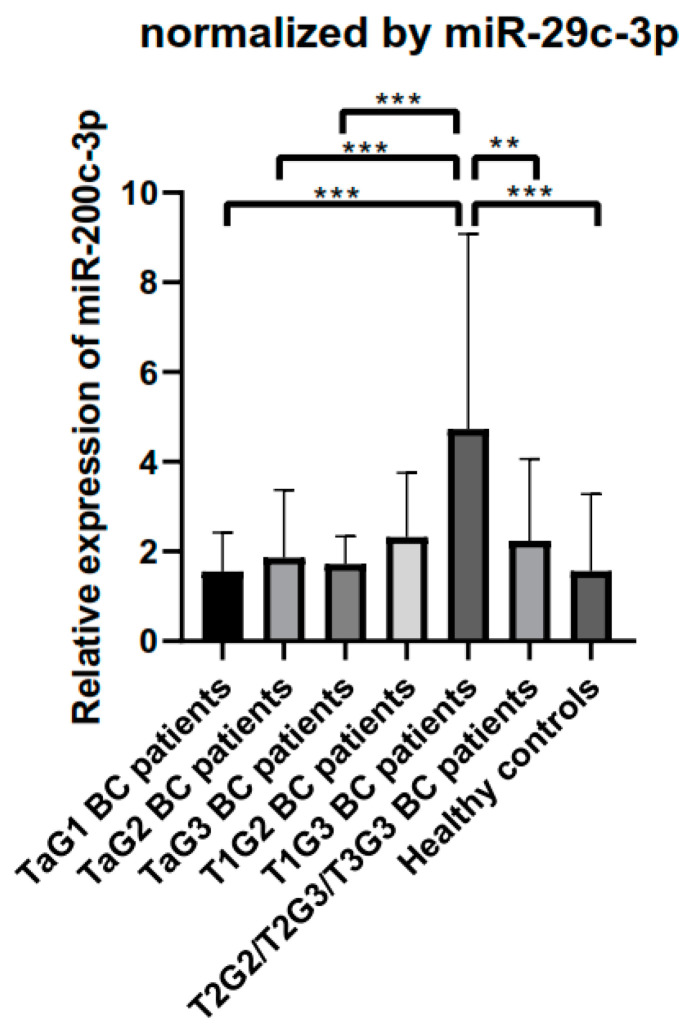
Relative expression of miR-200c-3p normalized by miR-29c-3p in the validation cohort. Normalization was performed by the 2^−∆∆Ct^ method. Error bars represent the standard error of the mean. ANOVA with the Tuckey Post Hoc test: ** *p* < 0.01; *** *p* < 0.001.

We verified the stability of miR-29c-3p in an independent cohort of BC patients and healthy controls. As in the screening cohort, no significant differences were observed in the expression of miR-29c-3p between BC patients (mean Ct = 27.65) and healthy controls (mean Ct = 28.13) (*p* = 0.37). Next we validated the robustness and efficacy of miR-29c-3p as endogenous control for BC in the validation cohort using the 2^−∆∆Ct^ method by analyzing the expression level of the BC-related miR-200c-3p in every group of BC patients and controls. As occurred in the samples studied in the screening stage, we observed significant differences in the expression of miR-200c-3p among the different clinical groups studied in the validation cohort (*p* < 0.001), with an increase of miR-200c-3p with the increase in BC stage (Figure 7). As occurred in the screening cohort, the biggest differences were found in the T1G3 BC group. As the patients with mildest stage of BC seem to have a lower expression level of miR-200c-3p, we grouped all the patients with the Ta stage and repeated the analysis. Figure S1a confirms that the expression of miR-200c-3p significantly increases with the severity of NMIBC, being T1G3 the BC type with the highest expression. Next, we grouped all NMIBC patients (TaG1+TaG2+TaG3+T1G2+T1G3) and compared the expression of miR-200c-3p to that of to MIBC patients (T2G2+T2G3+T3G3) and healthy controls. While NMIBC patients still showed the highest expression level of miR-200c-3p, no significant differences were observed when compared to MIBC or healthy controls (Figure S1b), probably because the highest difference occurs within the NMIBC group.

## 4. Discussion

New non-invasive markers are presently being under study to circumvent several drawbacks in BC diagnosis, monitoring and prognosis. Urine miRNAs are non-invasive promising biomarkers that have been previously proposed for BC diagnosis [13,14,15,16,17,19,20,21,23,24]. However, huge discrepancies arise among miRNA studies, to a great extent due to nonexistence of standardized procedures. Although the populations studied, sample processing, and RNA isolation and miRNA quantification methods are partly responsible for these inconsistencies; the normalization strategy used may represent the main hurdle. In fact, to minimize the effect occasioned by methodology-related factors on miRNA expression levels, an accurate data analysis ought to be performed using appropriate normalizers for external and internal variation correction [26]. These normalizers should be chosen from a selection of candidates that are expected to be stably expressed over the entire range of samples being investigated, since miRNAs can be affected by the condition under study. As an alternative, the mean expression value of all commonly expressed microRNAs in a given sample has been proposed for normalization [34]. Although this strategy presents good and robust results it implies that a large number of miRNAs have to be always profiled, which may not be possible or cost-effective in all studies [26,35]. Thus, as a general guideline suggested by the manufacturer, the use of the global mean for normalization is limited to the use of PCR panels that contain a larger number of microRNA assays, and it cannot be used for studies analyzing less than 20–50 different miRNAs.

Different RNA species have been proposed as normalizers (snRNAs, snoRNAs, rRNAs, miRNAs or exogenous synthetic RNAs); however, substantial differences in length and structure to that of miRNAs generate a high variability in the results [26]. Regarding the use of exogenous synthetic RNAs as normalizers, these are meant to track isolation and reverse transcription efficiency in order to eliminate deviations in the experimental process and make the results more reliable. However, it is important to remark that their use would never correct for deviations in sampling, in the quality of samples or in the amplification process. In fact, age, sample collection, preparation or storing can modify miRNA expression levels, which may be caused by cell lysis or miRNA degradation [26].

Several molecules have been proposed as normalizers for miRNAs studies in different disorders, mainly in blood [36], plasma [37,38,39], cell cultures studies [25,40], tissue [41] and urine [35,42,43,44]. However, there is no consensus, even in the same sample type, regarding which is the best normalizer for miRNA studies. Indeed, no consensus exists on a robust normalizer for urine studies. A recent study proposed miRNA miR-193a and miR-448 as normalizers [35]. However, in this study, only urine from healthy donors was analyzed and these results could vary when urine from cancer patients is investigated. In prostate cancer, miR-191-5p showed the lowest degree of variation and the highest stability value [42]. In our study, miR-191-5p ranked 29 out of 110 according to the comprehensive ranking of RefFinder, thus it is not a reliable normalizer for BC studies in urine. miR-16 was identified as the most stable endogenous reference miRNA in chronic kidney disease, making it a suitable normalizer for urinary exosome-derived miRNA [43]. Conversely, in our data set, miR-16-5p ranked 106 out of 110 according to the comprehensive ranking of RefFinder, turning it an ineffectual normalizer for urine BC studies. Finally, U6 has been proposed as normalizer for miRNA studies in urinary sediment of IgA nephropathy [44]. Although U6 was widely used as normalizer in countless studies at the origins of miRNA investigation, it is a member of the larger small RNA species which have a different biogenesis pathway (originate from the nucleus), may not be secreted or protected in cell-free biofluids in the same way that microRNAs are and may also behave differently during RNA isolation. Thus, U6 is an unreliable normalizer for urine miRNA studies. Other studies have employed the combination of two stable miRNAs as normalizers however, this strategy presents several drawbacks: it may increase technical variability, it implies a higher economic cost since the number of miRNAs to be quantified by RT-qPCR increases and it is more time consuming. Altogether, the use of a combination of miRNAs as normalizers hampers the direct translation of miRNA studies to daily clinical practice with diagnostic/staging purposes.

In the present study, we set for the first time the aim to ascertain the best miRNA normalizer for miRNA studies in urine of BC patients in order to avoid future inconsistencies among studies. We evaluated the performance of 110 candidate miRNAs with the comprehensive tool RefFinder that integrates 4 programs (Genorm, BestKeeper, Delta Ct method and NormFinder) in 35 BC patients and 15 healthy controls. We selected miR-29c-3p as the best normalizer for miRNA studies in urine of BC. It was the most stable miRNA according to the comprehensive analysis of RefFinder among the 110 studied, and also according to the Delta Ct method and NormFinder. Moreover, it had a good expression level in urine (mean Ct value in BC patients and healthy controls = 28.4) and no differences were observed between BC patients and controls, both in the screening and the validation cohorts.

Urine miR-200c-3p has been previously related to BC and it has been proposed as diagnostic and staging marker [24,33]. miR-200c-3p appears to control the epithelial-to-mesenchymal transition process through BMI-1 in BC cells, and it inhibits their proliferation by down-regulating E2F3 [45]. Thus, we selected this miRNA to test the robustness of miR-29c-3p as endogenous normalizer. We found significant differences in miR-200c-3p among the different clinical groups studied both in the screening and validation cohorts, with a trend in the increase of miR-200c-3p with the severity of NMIBC. The evaluation of additional stably expressed miRNAs proposed by RefFinder may have rendered other potential normalizers for urine miRNA studies in the context of BC, what represents a limitation of our study. Nonetheless, our results confirm previous findings and reinforce the use of miR-29c-3p as normalizer.

## 5. Conclusions

In summary, our study is the first report characterizing a reliable normalizer for the analysis of urine miRNAs in BC patients. miR-29c-3p, being one of the most stably expressed miRNAs in urine of BC patients and healthy individuals, arises as an optimal reference miRNA that may allow the comparison of future urine miRNA studies as non-invasive biomarkers for BC diagnosis and monitoring.

## Figures and Tables

**Table 1:** Clinical characteristics of the BC patients and healthy controls studied.

	BC Patients		Controls	
	Screening (*N* = 35)	Validation (*N* = 153)	Screening (*N* = 15)	Validation (*N* = 57)
**Age, *y***	67 (61–74)	69 (63–75)	64 (51–76)	64 (56–68)
**Male sex, *N (%)***	32 (91.43%)	129 (84.31%)	12 (80.00%)	43 (75.44%)
**Urine creatinine, *mg/dL***	76.5 (37.4–123.3)	77.4 (51.9–118.9)	78.5 (49.0–100.2)	98.2 (69.4–161.6)
**Tumor Stage and Grade, *N (%)***				
**TaG1**	10 (28.57%)	33 (21.57%)	-	-
**TaG3**	8 (22.86%)	13 (8.50%)	-	-
**T1G3**	5 (14.29%)	29 (18.95%)	-	-
**TaG2**	-	54 (35.29%)	-	-
**T1G2**	-	9 (5.88%)	-	-
**T2G2/T2G3/T3G3**	12 (34.29%)	15 (9.80%)	-	-

Continuous variables are presented as median and interquartile range and categorical variables are presented as count and percentage.

## Supplementary Materials

The following are available online at https://www.mdpi.com/2227-9059/8/11/447/s1, Table S1: miRNAs and internal controls included in the Serum/plasma miRNA PCR Panel V5 (Qiagen); Figure S1. Relative expression of miR-200c-3p normalized by miR-29c-3p in the validation cohort. (a) Comparison of the mildest stage of NMIBC patients (TaG1+TaG2+TaG3) and the other clinical groups. (b) Comparison of NMIBC patients (TaG1+TaG2+TaG3+T1G2+T1G3), MIBC (T2G2+T2G3+T3G3) and healthy controls. Normalization was performed by the 2^−∆∆Ct^ method. Error bars represent the standard error of the mean. ANOVA with the Tuckey Post Hoc test: * *p* < 0.05; ** *p* < 0.01; *** *p* < 0.001.
