# Supplementary material for: Identification of miR-29c-3p as a Robust Normalizer for Urine microRNA Studies in Bladder Cancer
Source: Biomedicines. 2020 Oct 22;8(11):447. doi: 10.3390/biomedicines8110447 (PMC7690381; doi:10.3390/biomedicines8110447)
Supplement: Supplementary file 1 [file biomedicines-08-00447-s001.zip › Figure S1.docx]

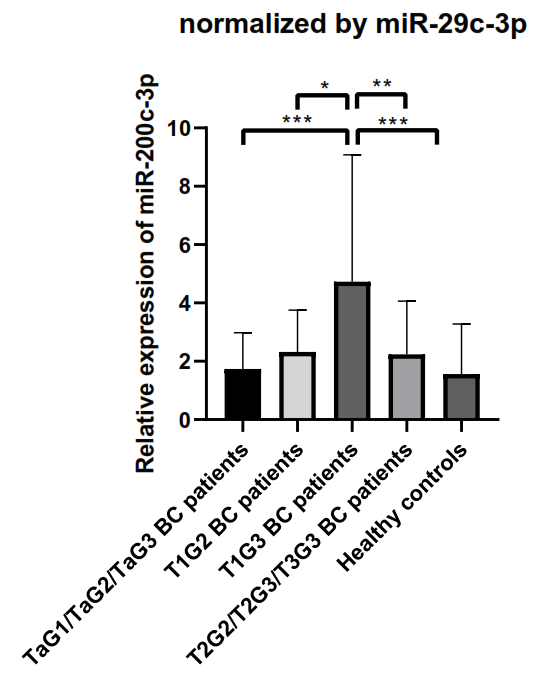


**a**

**b**


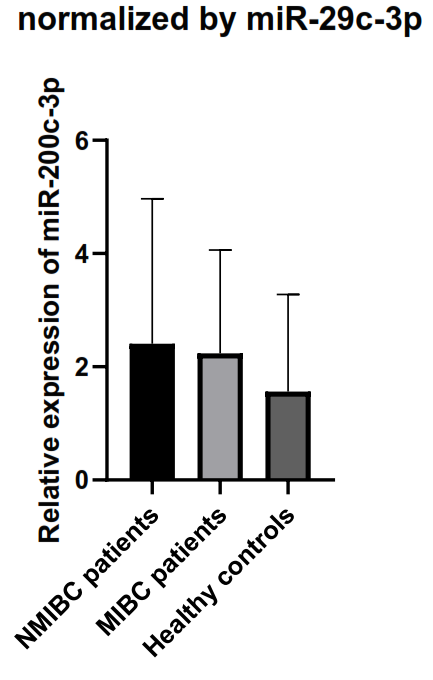


**Figure S1.** Relative expression of miR-200c-3p normalized by miR-29c-3p in the validation cohort. a) Comparison of the mildest stage of NMIBC patients (TaG1+TaG2+TaG3) and the other clinical groups. b) Comparison of NMIBC patients (TaG1+TaG2+TaG3+T1G2+T1G3), MIBC (T2G2+T2G3+T3G3) and healthy controls. Normalization was performed by the 2^–∆∆Ct^ method. Error bars represent the standard error of the mean. ANOVA with the Tuckey Post Hoc test: **P*<0.05; **, *P* < 0.01; ***, *P* < 0.001.
